# Complete Genome Sequence of Lactobacillus bulgaricus VHProbi R03, a Potential Fermentation Strain Isolated from Fermented Milk

**DOI:** 10.1128/mra.00875-22

**Published:** 2023-01-25

**Authors:** Kailing Li, Hongchang Cui, Zhi Duan

**Affiliations:** a Qingdao Vland Biotech Group Co., Ltd., Qingdao, China; Loyola University Chicago

## Abstract

The Lactobacillus bulgaricus strain VHProbi R03 is a novel starter culture that was isolated from naturally fermented milk. Whole-genome sequencing-based analysis is an ideal approach to elucidate the probiotic mechanism of action of this strain. Its genome contains a circular chromosome with 1,873,403 bp, and no plasmids exist in the genome.

## ANNOUNCEMENT

The species Lactobacillus bulgaricus is one of the predominant starter cultures in the dairy industry (1, 2). One milliliter of homemade fermented milk that had been collected from herdsman in Qinghai, China (97.36°E, 37.36°N), was serially diluted, spread on de Man-Rogosa-Sharpe agar (Haibo, China) plates, and then incubated under anaerobic conditions at 37°C for 48 h. A white colony designated VHProbi R03 was picked up and identified as the species Lactobacillus bulgaricus based on 16S rRNA gene Sanger sequencing.

A combined strategy with the Illumina HiSeq 2500 platform and Pacific Biosciences (PacBio) RS II sequencing technology was used to sequence the whole genome of L. bulgaricus VHProbi R03. The strain was cultured under the same conditions as used for isolation for DNA extraction. The total DNA was extracted using a genomic DNA purification kit (Promega, USA) following the manufacturer's instructions. Illumina template DNA was sheared into 400-bp fragments for library creation using a TruSeq library preparation kit from Illumina. The prepared libraries were then used for paired-end Illumina sequencing (2 × 150-bp reads). For PacBio sequencing, an aliquot of 15 μg DNA was centrifuged in a g-TUBE (Covaris, Woburn, MA) to obtain sheared fragments. DNA fragments were then purified, end repaired and ligated with SMRTbell sequencing adapters (PacBio, San Diego, CA) following the manufacturer’s recommendations, and sequenced on the PacBio RS II platform. Finally, 6,102,658 raw reads and 5,989,120 clean reads were generated, to reach a depth of 600.25-fold coverage, using the Illumina sequencing platform, and 1,237,87 raw reads (*N*_50_, 216,392 bp) were generated using the PacBio RS II sequencing platform. The Illumina raw reads were quality filtered with Sickle v1.33 (https://github.com/najoshi/sickle), and low-quality reads were removed before assembly. The PacBio raw reads were then assembled into a contig using Unicycler v0.4.8 (3) without quality control. Pilon v1.22 (4) was used for error correction of the PacBio assembly results. The final circularization step was checked manually; if there was an overlap at the two ends of the final assembly sequence with a certain length, then the sequence was looped and one end of the overlap sequence was cut off to obtain a circular sequence. Glimmer v 3.02 (5), tRNAscan-SE v2.0 (6), and barrnap v0.9 (https://github.com/tseemann/barrnap) were used to predict protein-coding genes, tRNAs, and rRNAs, respectively. CheckM v1.1.3 (7) was used to check the quality of the assembled genome sequence and indicated completeness of 99.03%, contamination of 0%, and strain heterogeneity of 0%. Genome annotation was conducted using the Prokaryotic Genome Annotation Pipeline (PGAP) v5.3 (8). Default parameters were used for all software unless otherwise specified.

The complete genome of VHProbi R03 contains a circular chromosome of 1,873,403 bp, with a GC content of 49.72% and 1,847 genes, 95 tRNA genes, and 27 rRNA genes. The Clusters of Orthologous Groups (COG) annotation revealed that 10.22% of the protein-coding genes were allocated to the category of amino acid transport and metabolism, which is greater than the annotated genes of Lactobacillus delbrueckii ACA-DC 87 (9). This finding may indicate that the strain of VHProbi R03 has greater potential in fermented yogurt applications.

### Data availability.

The complete genome sequence of L. bulgaricus VHProbi R03 has been deposited in GenBank with accession number CP096210, SRA accession numbers SRR18904155 and SRR18904156, BioProject accession number PRJNA831326, and BioSample accession number SAMN2772703.
